# A Rational Exposition of the Physical Signs of the Diseases of the Lungs, &c.

**Published:** 1834-01-01

**Authors:** 


**A RATIONAL EXPOSITION OF THE PHYSICAL SIGNS OF THE DISEASES OF THE LUNGS AND PLEURA; ILLUSTRATING THEIR PATHOLOGY AND FACILITATING THEIR DIAGNOSIS. By *Charles J. B. Williams*, M.D. Second Edition, 1833.**

THE second edition of Dr. Williams' valuable work on Auscultation having appeared, we deem it an advantage to our readers generally, and to those who hold the first edition particularly, to lay before them the substance of the new matter introduced into this edition. These additions are chiefly in the Sections on Percussion, and on the Auscultation of the Heart, which have been re-written. Dr. W. has also added an Appendix, in which are described various unsettled points connected with the latter subject.

The percussor or pleximeter which Dr. W. now uses, is a thin disk of ivory, about an inch in diameter, covered, on one side, with leather, and having, on the same side, two little projections, by which the fingers may press it firmly on the point of examination. We have lately used a flat piece of India-rubber, with all the advantage of the ivory—but we prefer the fingers.

"There is, however, a kind of mediate percussion requiring the aid of no instrument, yet so easy in its application, and accurate in its results, that it has generally superseded pleximeters on all common occasions. I allude to the use of the fingers of the left hand as a pleximeter. If one or more of these with the back outwards, be applied to any part

---

of the chest, percussion may be practised on them, without annoyance to the patient, and with the effect of eliciting a much louder sound than can be obtained by direct percussion. The pliancy and capability of the fingers, by which they can singly or collectively be made to fit any inequality in the surface of the chest, single fingers being used where delicacy is required, and all four where only a general survey of the chest is wanted, render this method so much more prompt and *handy*, that I have no doubt of its adoption, to the exclusion of other modes." 22.

Dr. W. justly remarks, that a person commencing the practice of percussion, will be guided more safely by the *comparative* than by the *absolute* sounds of different parts of the chest; and although he should lose no opportunity of acquainting himself with the sounds both of percussion and auscultation in healthy subjects, he should, in case of disease, direct his attention to irregularities or want of correspondence of sound in the two sides of the chest. The difference of sound in two corresponding parts of the same chest is often so striking, that the patient instantly remarks it, and is surprized at it.

In respect to auscultation of the heart, our author, in the first edition, expressed a distrust as to our knowledge of the signs produced by the motions of the heart—and later researches, he thinks, have proved the views of Laennec to be, in some respects, erroneous. He thinks the observations and opinions of Dr. Hope are better established than any others, and he therefore modifies the descriptions of Laennec by the practical conclusions of the author above-mentioned. But without repeating descriptions which have already been made, we shall give place to the Appendix itself, as containing many matters for the serious consideration of all auscultators. The extract is long; but it is very important, and insusceptible of analysis.

